# Acceptability of Telelactation Services for Breastfeeding Support Among Black Parents: Semistructured Interview Study

**DOI:** 10.2196/50191

**Published:** 2023-12-29

**Authors:** Khadesia Howell, Gabriela Alvarado, Molly Waymouth, Jill Demirci, Rhianna Rogers, Kristin Ray, Lori Uscher-Pines

**Affiliations:** 1 RAND Corporation Arlington, VA United States; 2 University of Pittsburgh Pittsburgh, PA United States

**Keywords:** acceptance, barrier, black parent, black, breastfeeding, concordance, consultant, consultation, digital divide, digital equity, disparity, ethnic, health equity, lactation, mother, parent, racial, telehealth, telelactation, user

## Abstract

**Background:**

While breastfeeding rates have increased in the United States in recent years, racial and ethnic disparities persist. Telelactation may help reduce disparities by increasing access to lactation consultants, but there is limited research on acceptability among minoritized individuals.

**Objective:**

We aimed to explore experiences with telelactation among Black parents and identify strategies to make services more culturally appropriate.

**Methods:**

We selected 20 Black parents who were given access to telelactation services from an ongoing National Institutes of Health–funded randomized controlled trial (the Tele-MILC trial) to participate in semistructured interviews. Interviews addressed birth experiences, use and opinions about telelactation, comparison of telelactation to in-person lactation support, and recommendations to improve telelactation services. The thematic analysis was informed by a previously reported theoretical framework of acceptability and RAND Corporation’s equity-centered model.

**Results:**

Users appreciated the convenience of telelactation and reported that lactation consultants were knowledgeable and helpful. Participants wanted more options to engage with lactation consultants outside of video visits (eg, SMS text messaging and asynchronous resources). Users who had a lactation consultant of color mentioned that racial concordance improved the experience; however, few felt that racial concordance was needed for high-quality telelactation support.

**Conclusions:**

While Black parents in our sample found telelactation services to be acceptable, telelactation could not, in isolation, address the myriad barriers to long-duration breastfeeding. Several changes could be made to telelactation services to increase their use by minoritized populations.

## Introduction

Exclusive breastfeeding (ie, breastfeeding without formula supplementation) is recommended by the American Academy of Pediatrics for at least 6 months post partum [1]. While national breastfeeding rates have increased in the United States in recent years, racial and ethnic disparities persist [2]. Breastfeeding rates tend to be lowest among young, low-income, Black, and unmarried parents, as well as parents with lower educational attainment and who participate in the Women, Infants, and Children (WIC) nutrition assistance program [3,4]. Despite overall increases in breastfeeding initiation in recent years, initiation continues to be lowest among Black parents (66.3% vs 84.3% among White parents) [4,5]. In addition, breastfeeding duration and exclusivity rates are lower among Black parents, with only 30% of Black infants receiving any breastmilk at 6 months, compared with 46% of White infants [6]. Lower breastfeeding rates, as seen among Black parents, result in poorer health outcomes for the birthing individual, child, and society more broadly [7,8].

Several studies have explored the underlying factors contributing to racial and ethnic breastfeeding disparities in the United States and pinpointed cultural differences and a lack of access to breastfeeding support as key drivers [3]. For example, barriers such as lack of social and cultural acceptance of breastfeeding and historical trauma particularly impact Black parents, whereas structural barriers such as maternal employment have been identified as more universal barriers to breastfeeding [3]. A qualitative study that explored factors influencing breastfeeding initiation among Black parents found that several complex, external factors contribute to low breastfeeding initiation, including racism, cultural beliefs about sexuality, the influence of familial networks, information sources, and other socioeconomic barriers [5].

Many studies have suggested that multilevel, integrative approaches are needed to help address the various breastfeeding barriers faced by Black parents [9]. For example, peer educator–led classes, peer counseling, home visits, and pre- and postpartum training have all been recommended to improve breastfeeding rates among Black parents [9]. Telelactation services, which connect breastfeeding parents with remotely located International Board–Certified Lactation Consultants (IBCLCs) through video visits, are another strategy that may support breastfeeding and potentially reduce disparities in breastfeeding. While some research has evaluated the effectiveness of these interventions, few studies have been conducted to date that explore how telelactation services impact breastfeeding rates among Black parents and whether the services provided are culturally appropriate.

Telelactation services vary based on a variety of characteristics, including whether visits are scheduled in advance versus available on demand or whether patients select the IBCLC they will see rather than being assigned to the first available IBCLC. Further, some telelactation providers charge per visit, while others use a membership model with unlimited visits [10]. Telelactation services have been made available in recent years to hundreds of thousands of parents of color through WIC programs, state and local public health entities, and Medicaid-managed care plans [10].

Although telelactation could help reduce disparities in breastfeeding rates by increasing access to IBCLCs, there is very little research on whether these services are culturally appropriate and meet the needs of Black parents. As with any innovation in health care delivery, there are risks associated with the introduction of a new service, including the possibility that it could inadvertently increase disparities if not implemented in a manner that centers on equity. In this study, we aimed to understand the experiences of Black birthing people with telelactation and to develop recommendations to improve telelactation services by focusing on the needs of this population.

## Methods

### Study Population and Sampling

We recruited 20 participants who were participating in a digital randomized controlled trial on the effectiveness of telelactation services (ClinicalTrials.gov NCT04856163). In the main trial, we advertised on pregnancy mobile phone apps with millions of users to recruit over 2000 primiparous, pregnant individuals aged 18 years and older who intended to breastfeed. Details on the trial design and methods are published elsewhere [11].

We selected interview participants from the trial who had self-identified as Black, received access to telelactation services through the trial (intervention arm only), and were between 8 and 12 weeks post partum. We chose this time frame to allow participants sufficient time to gain experience with telelactation services and to minimize recall bias. We used heterogeneity sampling to ensure that the final sample varied with respect to US state, socioeconomic status, and use of telelactation. Participants were given a US $40 Amazon gift card as an incentive. Participants completed informed consent upon enrollment in the trial and provided verbal informed consent at the time of the interview.

**Figure 1:**
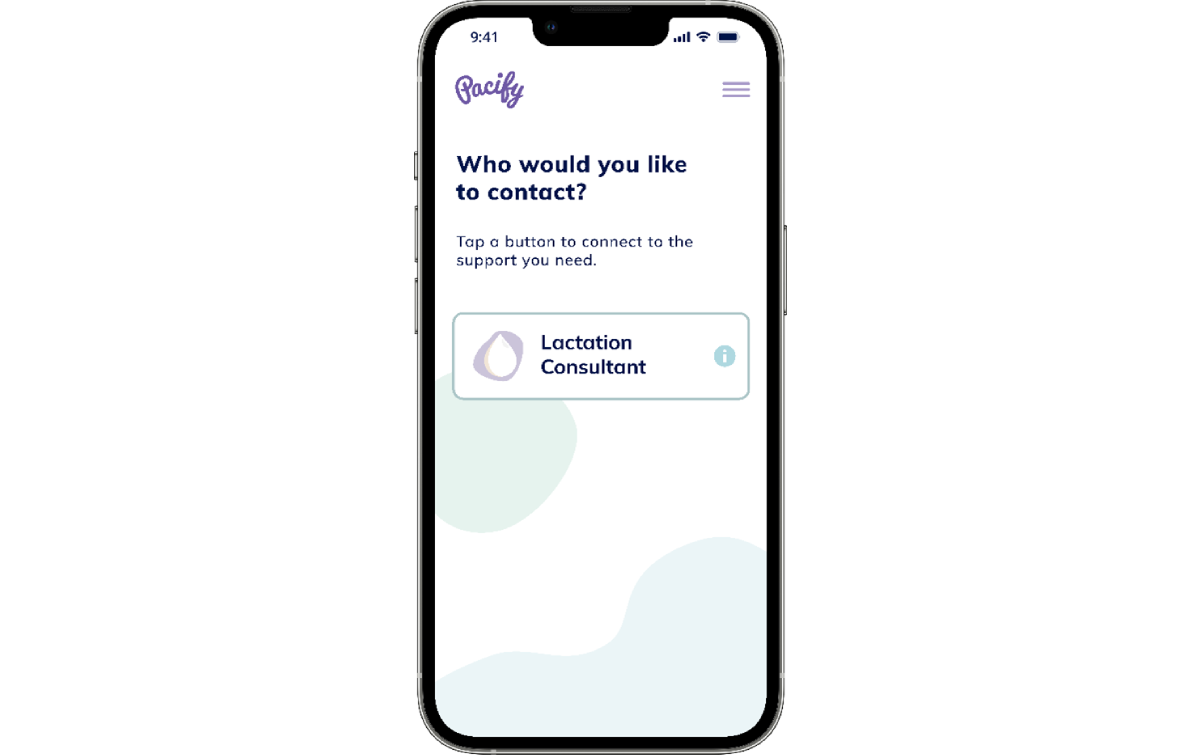
View of the telelactation app.

The telelactation services that participants received were provided by Pacify Health. Participants downloaded the app, which provides on-demand, unscheduled video visits with IBCLCs. IBCLCs are available 24 hours a day (Figure 1). Although all participants in the intervention arm of the trial were given access to telelactation, participants ultimately determined whether and how to engage with it.

Interviews were conducted by the first author of the paper, who identifies as a Black American, cisgender woman. The rest of our team included researchers who identify as Latinx, mixed race, and White. The research team is multidisciplinary and includes physicians, nurses, lactation specialists, health services researchers, clinical researchers, and public health and policy researchers. A total of 3 members of the team are trained in health equity and 5 in qualitative methods.

This study was informed by the equity-centered model developed by the RAND Corporation’s Center for Advancing Racial Equity Policy (CAREP) [12]. Key principles from the CAREP model were incorporated in several ways (Textbox 1).

**Model construct and study application of RAND Corporation’s equity-centered model.**

**Reflexivity**

- We consistently reflected on how the research team’s lived experiences and assumptions impacted the study. Multiple members of the study team had first-hand experiences with breastfeeding and receiving or providing breastfeeding support. The lead author stated her position at the beginning of each interview to build trust and rapport.

**Holistic contextualization**

- We elicited perspectives about parents’ complete breastfeeding experience (from their birth experiences to their experiences with telelactation) through narratives.

**Action-oriented outcomes**

- We explicitly asked participants for their recommendations on how to improve telelactation services rather than making assumptions about how services could better meet the needs of Black parents.

### Data Collection

Interviews were conducted from September to December 2022 and followed a semistructured guide. Questions addressed the following topics: (1) cultural, family, and community influences on breastfeeding; (2) breastfeeding experiences; (3) sources of breastfeeding support; and (4) experiences with telelactation. Questions on experiences with telelactation differed depending on whether the interviewee was a user or nonuser of the telelactation app. Interviews were recorded and transcribed. Recruitment occurred until we reached thematic saturation, defined as the point at which additional interviews did not generate new themes or change our characterization of existing themes.

### Analysis

We developed a codebook that was informed by questions included in the interview guide as well as domains of Sekhon et al’s [13] theoretical framework of acceptability. This framework suggests that for a health care intervention to be effective, it must first be acceptable to a population. Acceptability consists of 7 constructs: affective attitude, burden, perceived effectiveness, ethicality, intervention coherence, opportunity costs, and self-efficacy [13].

The codebook was refined through piloting on an initial interview transcript and consensus-building sessions with the study team. To ensure interrater reliability, 2 coders (KH and LUP) engaged in an initial training phase where they collaboratively coded 1 transcript in real time and refined the codebook definitions to achieve alignment. After this phase, they blind-coded 2 transcripts and conducted an interrater reliability test in Dedoose (version 9.0.82; SocioCultural Research Consultants), a qualitative analysis software. They achieved a score of κ=0.71, which suggests substantial alignment. After this process was complete, the 2 coders independently coded the remaining 17 transcripts using Dedoose.

KH and LUP organized codes under Sekhon et al’s [13] constructs with supporting quotes. The code organization was discussed and finalized in weekly meetings with the study team. We reported all methods and results according to the Standards for Reporting Qualitative Research (SRQR) Guidelines.

### Ethical Considerations

RAND’s institutional review board approved the study (2020-N0641), with the informed consent being verbal. This verbal consent was obtained at the beginning of each interview. The approval also covered any type of analysis that was needed (primary and secondary data). All data were deidentified throughout the study. Participants were compensated US $25 for their participation in this part of the study.

## Results

A total of 20 Black parents representing 12 different states participated in the interviews. Of which, 13 (65%) participants used the telelactation services provided by the study to obtain breastfeeding support, and 7 (35%) chose not to use telelactation for various reasons. A total of 10 (50%) participants had a household income of less than US $54,999 per year (Table 1).

Below, we describe our findings organized by Sekhon et al’s [13] constructs, which are also summarized in Table 2. We did not address opportunity costs because telelactation was provided free of charge through the study and was considered a low-burden intervention.

**Table 1:** Participant characteristics.

Participant characteristic		Value, n (%)
**Use of telelactation**		
	User	13 (65)
	Nonuser	7 (35)
**Region**		
	Northeast	7 (35)
	Southeast	8 (40)
	West	2 (10)
	Midwest	3 (15)
	Southwest	0 (0)
**Household income (US $)**		
	<25,000	4 (20)
	25,000-54,999	6 (30)
	>55,000	8 (40)
	Unknown	2 (10)
**Number of telelactation visits completed**		
	0	7 (35)
	1	8 (40)
	2	4 (20)
	>3	1 (5)
**Breastfeeding at time of interview**		
	Yes	11 (55)
	No	9 (45)

**Table 2:** Telelactation findings by Sekhon et al’s [13] theoretical framework of acceptability.

Theoretical framework of acceptability	Telelactation constructs	Main themes
Affective attitude	Feelings about telelactation	Users and nonusers had positive views of telelactation. Telelactation was perceived as easy to use and convenient.
Burden	Burden of telelactation	Telelactation was less burdensome than in-person support. Telelactation posed other burdens in terms of physical appearance and image.
Ethicality	Telelactation aligns with values	Some users valued seeing an IBCLC^a^ of the same race or cultural background. Users valued building relationships with their IBCLC; telelactation did not always allow for connections with the same IBCLC for repeat visits.
Intervention coherence	Understanding and usability of telelactation.	Telehealth was perceived as easy to use and intuitive. Some participants were not sure the service would actually be available 24/7 or had misgivings about initiating a video call late at night.
Opportunity costs	Which benefits, values, profits, etc must be given up to participate in the intervention?	Not applicable
Perceived effectiveness	The extent to which the intervention likely achieved its purpose.	Users provided numerous examples of receiving practical advice that changed their approach to breastfeeding. While many participants perceived telelactation to be effective, several also pointed out that telelactation was likely less effective than in-person care. Nevertheless, the convenience of telelactation made up for the (somewhat) reduced effectiveness.
Self-efficacy	Individuals can perform the behavior required for the intervention.	Participants generally expressed confidence in using telelactation services.

^a^IBCLC: International Board-Certified Lactation Consultant

### Feelings About Telelactation

The first domain of the acceptability framework focuses on how individuals feel about a particular intervention. Participants, including both users and nonusers of telelactation, generally had positive views of the service. Multiple participants felt that the service was easy and straightforward to use. Further, they appreciated the convenience of receiving services from home and the around-the-clock availability of IBCLCs.

I think what I did like about the app was that they [the IBCLCs] were 24/7.

### Burden of Telelactation

The second domain of the acceptability framework focuses on the time and effort it takes to participate in an intervention. Participants generally agreed that one of telelactatation’s best features is that it is less burdensome than seeking in-person support. Seeking in-person support not only requires more time but also presents challenges related to transportation and traveling with an infant.

I think I really like being in the comfort of my home and getting advice, because going out with him [my baby] because I don't really have help. I am a single mom and I don't really have family…I really have to take him [my baby] everywhere I go.

The perceived need to look presentable for a video visit was framed as a burden for some parents, and concerns over image discouraged a handful of parents from using telelactation services.

I probably had a little bit of reservations because when you do a video call, you don't wanna look crazy…I think that when I had-, didn't call the last time, it was because I just didn't feel like getting up and making myself look somewhat decent.

For several reasons, including concern about the image and awkwardness of doing a video call with an unknown provider, multiple participants recommended that telelactation services offer additional ways for parents to engage with IBCLCs beyond video visits. For example, some requested written resources because they “didn’t always want to talk to someone” or texting options that could be converted to video visits if needed.

I would definitely do the text. Sometimes I want to talk to someone, but I don't want to be face-to-face or actually talking. It's just easier, especially when I have the baby.

### Ethicality of Telelactation

The third domain of the acceptability framework focuses on whether an intervention is a good fit with an individual’s value system. Within this domain, a few participants spoke about the value of seeing an IBCLC of the same race or cultural background and noted that it improved the quality of the visit.

I was excited and I felt more at ease opening up and saying what was really going on because there was somebody [IBCLC on telelactation] that looked like me. And not to even be culturally biased, it makes a difference, especially because I had a very traumatic birth...So it was very comforting and it made me more apt to use it [telelactation] again because if somebody would've told me, oh no, and been demeaning and what I received in the hospital, I probably would've just never even thought about [telelactation] again.

However, the importance of racial alignment in telelactation visits was not universal.

I don't think the rest [race] really plays a major part when it comes to lactation, but it does play a part when it comes to the actual birth process.

A few participants noted that they valued building personal relationships and rapport with their IBCLC, and they did not like the fact that with this telelactation service, the participant was unlikely to get the same IBCLC if they requested another visit. Several participants recommended that the service have the capability to connect them to the same IBCLC for repeat visits.

### Coherence of Telelactation

The fourth domain of the acceptability framework focuses on an individual’s understanding of the intervention and how it works. Participants generally felt that the service was easy to use and intuitive.

Yeah, overall, it was a pretty easy process. Just open the app and there's just one button to click and it just started trying to connect me with somebody.

Although the telelactation service was available around the clock, some participants were not sure the service would actually work or had misgivings about initiating a video call in the middle of the night (ie, because they did not want to inconvenience an IBCLC).

[I didn’t use the service] because sometimes I have a question really late in the night and I'm not sure if someone's going to pick up. And then at some points in the day I'm not dressed, or I just feel unprepared.

### Perceived Effectiveness of Telelactation

The sixth domain of the acceptability framework focuses on the extent to which the intervention is likely to achieve its purpose. Participants agreed that the IBCLCs that they interacted with were knowledgeable and helpful, and they provided numerous examples of receiving practical advice that changed their approach to breastfeeding.

Both [IBCLCs I talked to on the app] were knowledgeable. They were both really sweet and just really wanted to help me…. the first time I had issues because of the clogged ducts, and then the other time, my issue was that I just wanted to stimulate more milk production. Both times when I was assigned to the lactation consultants, both of them knew those areas well and were able to assist me with those issues.

While many participants perceived telelactation to be effective, several also pointed out that telelactation was likely less effective than in-person care. Nevertheless, the convenience of telelactation made up for the (somewhat) reduced effectiveness.

In-person was definitely better. And like I said, she [the IBCLC who provided telelactation] was nice. But when I had everything in person it is just we had longer conversations. They could actually see what I was doing...I think convenience is also really important. That's why I would use it [telelactation] again.

### Self-Efficacy

The seventh domain of the acceptability framework focuses on the extent to which an individual has confidence that they can perform the behaviors required to participate in an intervention. Participants generally expressed confidence in using telelactation services; however, there were some examples of participants noting that they “didn’t have enough time to do a [video] call without interruptions” or were uncertain if they needed support from family members (eg, to hold the baby) when using telelactation. Several participants made the point that, although they were confident about using telelactation, the service was not sufficient to overcome the myriad barriers to breastfeeding that parents face.

*I mean they all pretty much would tell me the same thing. So, they was telling me things that I was already doing and that I was already taught. When you're tired, you've got a lot going on…Maybe if I had help where I didn't have so much going on… But I felt like I did the best I could and I reached out to as much people as I could, and I wasn't getting anywhere with it….* *They don't know if I have time for that...I'm in the house and I help my granddad. I'm taking care of my baby…. And as far as me being able to do certain things and the resources they're telling me, I just don't have time for that. And I barely have time to get myself right.*

## Discussion

### Overview

Sekhon et al’s [13] framework theorizes that acceptability is a multifaceted construct that can be assessed through behavior and cognitive and emotional experiences. Telelactation services were acceptable and convenient for Black parents in our sample. Participants valued the service because it removed some of the barriers to in-person care (eg, transportation). However, connecting to IBCLCs through video was uncomfortable for some participants (eg, because of the perceived need to look presentable). Furthermore, some participants wanted options to engage with IBCLCs beyond video visits. Previous work has shown that telelactation is feasible and acceptable among predominantly White parents who face barriers to in-person breastfeeding support [14-16]. This research demonstrated that the same was true for Black parents, and, thus, telelactation services with or without video may be one avenue to reduce racial disparities in breastfeeding rates, especially if tailored to address specific barriers voiced in this study.

Although not universal, several participants shared that they valued a shared racial identity with their IBCLC. Studies have shown that racial and cultural concordance can result in more positive health behaviors, beliefs, and outcomes [17,18]. For example, a systematic review of 40 studies found that racial concordance is consistently associated with better communication [19]. Another study compared information-seeking behavior following receipt of targeted health messaging and found that racially aligned physicians were perceived as more trusting sources of information among Black participants [20]. These studies and our findings suggest that the ability to receive breastfeeding support from a racially concordant IBCLC can be valuable and effective for some Black parents.

The equity-centered model we used in the design and conduct of this study prioritizes action-oriented outcomes that are community-driven (ie, not based on researchers’ assumptions). Based on this model, we identified the following recommendations to improve experiences with, and perhaps the demand for, telelactation services among Black parents. First, given that some parents want options to engage with IBCLCs without the burden of having to prepare for and conduct video visits, telelactation services could add texting features and allow parents to begin calls as audio-only visits that can be converted to video at the request of the IBCLC or parent. With or without these features, telelactation services or individual IBCLCs could present parents with a psychological safety statement (eg, address video recording). In addition, given that some parents value seeing IBCLCs with a shared cultural, racial, or ethnic identity, services could consider ways to accommodate these preferences (eg, give parents the option to be notified when a specific IBCLC is available for less urgent needs). Finally, as the landscape of telelactation evolves, it is imperative to continue to center marginalized communities’ voices to improve quality of care and support equitable access and outcomes.

### Limitations

This study had several limitations. We did not engage all stakeholders who are affected by the implementation of telelactation programs. While telelactation providers can learn from our findings, the principle of holistic contextualization suggests that future research should engage additional groups as well. Second, we only explored experiences with one telelactation model. Third, although we continuously reflected on the role of intersectionality in breastfeeding and the receipt of breastfeeding support, given our sample size, we were not able to describe the roles of socioeconomic status, gender identity, etc in participants’ perceptions and experiences.

### Conclusions

This study provides insight into Black birthing people’s experiences with telelactation. Given the acceptability of telelactation in our sample, it has the promise to improve access to professional breastfeeding support among Black parents and possibly other historically excluded racial and ethnic groups. Nonetheless, Black parents also spoke of numerous, complex barriers to breastfeeding that telelactation in isolation could not overcome. These barriers included a lack of support systems, limited access to various health care services, and negative experiences with health care providers that impacted interest and confidence in health care services more broadly. Thus, telelactation fills a somewhat narrow role and may be regarded as one component of a comprehensive effort to increase access to professional support and reduce disparities in breastfeeding rates.

## Abbreviations

CAREP: Center for Advancing Racial Equity Policy
IBCLC: International Board-Certified Lactation Consultant
SRQR: Standards for Reporting Qualitative Research
WIC: Women, Infants, and Children
